# Associations of short stature and components of height with incidence of type 2 diabetes: mediating effects of cardiometabolic risk factors

**DOI:** 10.1007/s00125-019-04978-8

**Published:** 2019-09-09

**Authors:** Clemens Wittenbecher, Olga Kuxhaus, Heiner Boeing, Norbert Stefan, Matthias B. Schulze

**Affiliations:** 1 Department of Molecular Epidemiology, German Institute of Human Nutrition Potsdam-Rehbruecke, Arthur-Scheunert-Allee 114-116, 14558 Nuthetal, Germany; 2 German Center for Diabetes Research (DZD), München-Neuherberg, Germany; 3 Department of Epidemiology, German Institute of Human Nutrition Potsdam-Rehbruecke, Nuthetal, Germany; 4 Department of Internal Medicine, Division of Endocrinology, Diabetology, and Nephrology, University of Tübingen, Institute for Diabetes Research and Metabolic Diseases of the Helmholtz Centre Munich at the University of Tübingen, Tübingen, Germany; 5 Institute of Nutritional Sciences, University of Potsdam, Nuthetal, Germany

**Keywords:** Adult height, Blood pressure, Diabetes incidence, Leg length, Liver fat, Short stature, Trunk length

## Abstract

**Aims/hypothesis:**

This study aimed to evaluate associations of height as well as components of height (sitting height and leg length) with risk of type 2 diabetes and to explore to what extent associations are explainable by liver fat and cardiometabolic risk markers.

**Methods:**

A case-cohort study within the European Prospective Investigation into Cancer and Nutrition (EPIC)-Potsdam study comprising 26,437 participants who provided blood samples was designed. We randomly selected a subcohort of 2500 individuals (2029 diabetes-free at baseline and with anamnestic, anthropometrical and metabolic data for analysis). Of the 820 incident diabetes cases identified in the full cohort during 7 years of follow-up, 698 remained for analyses after similar exclusions.

**Results:**

After adjustment for age, potential lifestyle confounders, education and waist circumference, greater height was related to lower diabetes risk (HR per 10 cm, men 0.59 [95% CI 0.47, 0.75] and women 0.67 [0.51, 0.88], respectively). Leg length was related to lower risk among men and women, but only among men if adjusted for total height. Adjustment for liver fat and triacylglycerols, adiponectin and C-reactive protein substantially attenuated associations between height and diabetes risk, particularly among women.

**Conclusions/interpretation:**

We observed inverse associations between height and risk of type 2 diabetes, which was largely related to leg length among men. The inverse associations may be partly driven by lower liver fat content and a more favourable cardiometabolic profile.

**Electronic supplementary material:**

The online version of this article (10.1007/s00125-019-04978-8) contains peer-reviewed but unedited supplementary material, which is available to authorised users.

## Introduction

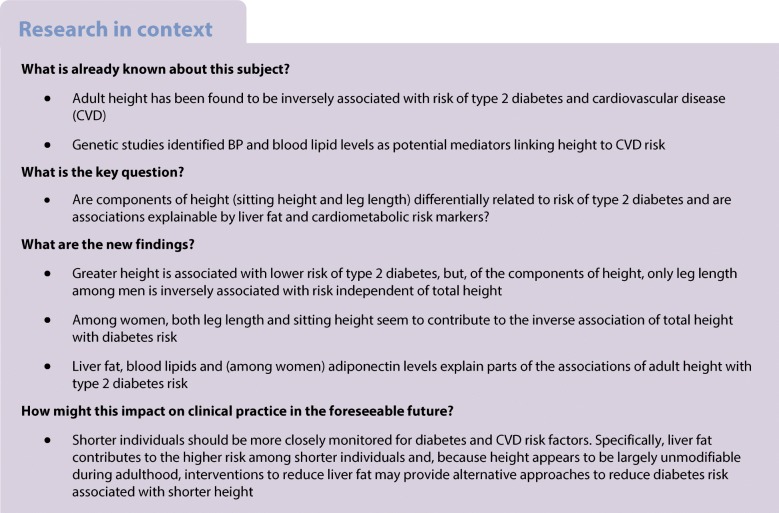



Short stature has been linked to higher risk of diabetes in prospective cohort studies, including the European Prospective Investigation into Cancer and Nutrition (EPIC)-Potsdam study [1, 2]. A meta-analysis of cohort studies reported an RR for comparison of highest and lowest categories of height of 0.85 (95% CI 0.76, 0.96), with a slightly stronger association in women (RR 0.83) compared with men (RR 0.87) [3]. Short stature is also related to higher cardiovascular risk [4] and measurement of height can be used for the prediction of diabetes alongside other risk factors [5, 6]. Height can be subdivided into the components sitting height and leg length. The latter has been linked with environmental and nutritional exposures during prepubertal growth periods [7]. However, only a few prospective studies have investigated the different components of height with regard to diabetes risk [8, 9].

The mechanisms of how height is associated with diabetes risk are largely unknown. Supporting a role for diabetes, it has been reported that taller people are more insulin sensitive and have better beta cell function [10–12], which might partly be a result of less ectopic fat storage (e.g. in the liver) [4]. Recent Mendelian randomisation studies support that height is associated with cardiovascular risk and that this risk might at least in part be mediated by cardiometabolic risk factors relevant for type 2 diabetes, namely BP, blood lipids and inflammation [13, 14]. However, the relevance of liver fat and cardiometabolic risk factors as potential mediating factors linking height and its components to diabetes risk remains largely unknown.

The objective of this study was to evaluate associations of height as well as components of height (leg length and sitting height) with risk of type 2 diabetes and to explore to what extent these associations are mediated by blood lipids, BP, C-reactive protein (CRP) and other markers related to liver metabolism and ectopic fat accumulation.

## Methods

### Study population and baseline measurements

The EPIC-Potsdam study includes 27,548 participants, 16,644 women aged mainly 35–65 years and 10,904 men aged mainly 40–65 years, from the general population of Potsdam, Germany, recruited between 1994 and 1998 [15]. Informed consent was obtained from all participants, and approval was given by the Ethical Committee of the State of Brandenburg, Germany. The baseline examination included anthropometric and blood pressure measurements, a personal interview and a questionnaire on prevalent diseases and sociodemographic and lifestyle characteristics, and a validated semi-quantitative food frequency questionnaire. The anthropometric measurement procedures and measures of quality control were previously described in detail [16, 17]. Briefly, body weights were measured by electronic digital scales, accurate to 100 g, with participants wearing only light underwear and after emptying the bladder. Total body height and sitting height were measured to the nearest 0.1 cm using a flexible anthropometer. We calculated leg length as the difference between total and sitting height. Waist circumference was measured midway between the lower rib margin and the superior anterior iliac spine to the nearest 0.5 cm with a non-stretching tape applied horizontally and the proper use controlled by a mirror. Measurement of BP was performed in the sitting position with the arm elevated at heart level using oscillometric devices (BOSO-Oscillomat, Bosch & Sohn, Jungingen, Germany) and the mean of the second and third reading was used [18].

For efficient molecular phenotyping, a nested case-cohort was constructed. With this type of study design, the results are expected to be generalisable to the source population without the need to measure biomarker levels in the entire cohort [19]. Based on the 26,437 participants who gave blood, a subcohort of 2500 participants (about 10%) was randomly drawn as being representative of the full cohort, and all incident diabetes cases were included (*n* = 820). After exclusion of participants without available plasma samples, 2483 participants in the subcohort and 798 incident cases were the basis for biomarker measurements. Excluding prevalent diabetes, self-reported diabetes or diabetes medication at baseline and participants with missing follow-up left 2307 members of the subcohort and 797 incident cases (overlap *n* = 74). For investigations of potential mediating factors, 2662 participants (2029 subcohort members, 698 cases, overlap *n* = 65) remained after exclusions for missing biomarker information or BP measurements.

### Ascertainment of type 2 diabetes

Follow-up questionnaires have been administered every 2 to 3 years with response rates of 96%, 95%, 91% and 90% in follow-up rounds 1, 2, 3 and 4 (by August 2005). Systematic information sources for incident cases were self-reports of a type 2 diabetes diagnosis, diabetes-relevant medication and dietary treatment due to type 2 diabetes during follow-up. Furthermore, we obtained additional information from death certificates or from random sources, such as the tumour centres, physicians or clinics that provided assessments from other diagnoses. Once a participant was identified as a potential case, disease status was further verified by sending a standard inquiry form to the treating physician. Only physician-verified cases with a diagnosis of type 2 diabetes (International Classification of Diseases, 10th revision code: E11; http://apps.who.int/classifications/icd10/browse/2016/en) and a diagnosis date after the baseline examination were considered confirmed incident cases of type 2 diabetes.

### Biomarker measurements

Venous blood (30 ml) was collected by qualified medical staff in a standardised procedure and subsequently fractioned, and plasma was stored in tanks of liquid nitrogen (approximately −196°C) or deep freezers (−80°C). The automatic ADVIA 1650 analyser (Siemens Healthcare, Erlangen, Germany) was used to assess plasma levels of total cholesterol, HDL-cholesterol, triacylglycerols and CRP; erythrocyte levels of HbA_1c_; and activity of γ-glutamyl transferase (GGT). Total plasma adiponectin concentrations were measured using an ELISA (Linco Research, St Charles, MI, USA). Blood samples were drawn in monovettes containing citrate as anticoagulant. Therefore, plasma concentrations were multiplied by 1.16 for women and 1.17 for men in order to receive levels for these citrate plasma samples comparable with levels obtainable from EDTA plasma [20]. We calculated the fatty liver index (FLI) consisting of BMI, waist circumference, GGT and triacylglycerols according to Bedogni et al [21].

#### Statistical analyses

We modelled height and its components (leg length and sitting height) as continuous variables. Furthermore, relative leg length was calculated as the leg length/height ratio expressed as a percentage. Association between height and its components and diabetes risk was evaluated in Cox proportional hazards regression models with age as underlying time scale. Study exit was determined by diagnosis of diabetes, dropout or censoring time, whichever came first. The case-cohort design was accounted for by Prentice weighting [19]. Potential confounders to be included as covariates were age (models stratified according to age at recruitment), waist circumference (cm), activity (sports [h/week], biking [h/week]), smoking (never a smoker, former smoker, current smoker), education (no vocational training or vocational training, technical school or technical college, university) and daily intake of alcohol (≤6 g/day, >6–12 g/day, >12–24 g/day, >24–60 g/day, >60 g/day [for women only], >60–96 g/day, >96 g/day [for men only]).

Because physical growth will not only affect height but also waist circumference, adjustment for waist circumference could be considered an overadjustment. We therefore attempted to adjust in sensitivity analyses only for the component of waist circumference that was not the result of a natural increase in waist accompanying body growth. For the latter, we ran sex-specific linear regression models separately for men and women with normal weight (BMI between 18.5 and 24.9 kg/m^2^) to predict expected waist circumference for a given height. This estimate was subtracted from the waist circumference measured and the obtained difference used for adjustment.

In mediation analysis, attenuation of the association between height and diabetes risk after adjustment for the FLI, BP and BP medication and for biomarkers was evaluated by comparing Cox models without and with adjustment for cardiometabolic risk factors. The statistical significance of attenuation was evaluated with a method introduced by Hoffmann et al [22], applying a one-sided Wald test (H_0_, beta-coefficient for height from the corresponding reference model ≤ beta-coefficient for height from the biomarker-adjusted model). The relative change of association and its stability as well as the corresponding HRs were estimated as median and dispersion from a bootstrapping procedure (500 bootstrap replicates) [23].

## Results

**Fig. 1:**
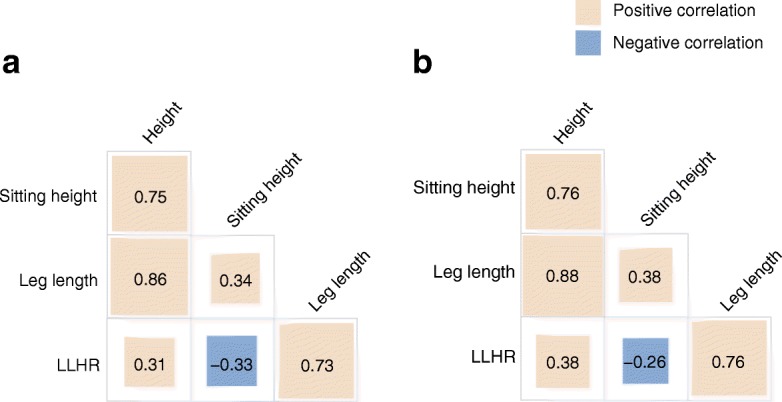
Age-adjusted correlations among height and components of height in a random subcohort (*n*=2029) of the EPIC-Potsdam study. (**a**) Men and (**b**) women. LLHR, leg length/height ratio

The correlations among height, leg length, sitting height and the leg length/height ratio are depicted in Fig. 1. Total height was positively correlated with both leg length and sitting height, although the correlation was stronger for leg length, with similar correlation coefficients by sex. The leg length/height ratio was only moderately positively correlated with height but inversely with sitting height.

Table 1 presents baseline characteristics in the random subcohort by quintiles of height for men and women. Taller men were generally younger, more likely to have a university degree, more likely current smokers and consumed more alcohol compared with shorter men. Such differences were less pronounced among women, but still, taller women were more likely to be better educated and to smoke compared with women of shorter stature. Height and all height components showed slight inverse correlations with FLI and cardiometabolic biomarker levels when adjusted for age and waist circumference (Table 2).

**Table 1:** Baseline characteristics of a random subcohort (*n* = 2029) of the EPIC-Potsdam study according to quintiles of height stratified by sex

Characteristic	Men (*n*=765)					Women (*n*=1264)				
	Quintiles of height (cm)					Quintiles of height (cm)				
	<169.7	169.7−<173.3	173.3−<176.4	176.4−<180.3	≥180.3	<157.8	157.8−<161.5	161.5−<164.5	164.5−<168.1	≥168.1
	*n* = 161	*n* = 145	*n* = 160	*n* = 144	*n* = 155	*n* = 247	*n* = 250	*n* = 238	*n* = 267	*n* = 262
Age (years)	56.3 (12.3)	53.5 (13.8)	53.5 (14.4)	51.2 (14.3)	47 (12.8)	52.9 (15.3)	49.1 (15.9)	48.9 (17.9)	46.1 (16.5)	43.4 (16.1)
BMI (kg/m^2^)	26.9 (3.8)	26.2 (4.5)	26.5 (5.2)	26.8 (4.9)	25.7 (4.1)	25.9 (5.9)	24.4 (5.2)	24.9 (5.1)	24.2 (5.6)	24.2 (5.4)
Waist circumference (cm)	92 (9)	92.5 (12)	94 (15)	94 (13)	94 (12)	79.5 (14.5)	76.5 (14)	78 (15)	77.5 (16)	79 (14)
Education (%)										
Technical college	12.4	15.9	16.9	16.7	12.3	26.7	32.8	29.8	29.6	29.8
University	42.2	51	51.9	57.6	58.1	22.3	25.2	29	35.6	37.4
Smoking status (%)										
Former	53.4	40	45	36.8	40.6	23.5	22.4	24.8	27.3	25.6
Current	16.8	30.3	28.1	30.6	27.1	15.4	16	19.3	15	20.6
Alcohol (g/day)	16.5 (21.3)	17.3 (23.7)	18.8 (23.5)	16.2 (26)	20.6 (24.2)	3.8 (8)	4.4 (8.7)	4.9 (10)	5.3 (8.5)	6.3 (9)
Sport (h/week)	0 (1)	0 (1)	0 (1.5)	0 (2)	0 (2)	0 (2)	0 (1)	0 (2)	0 (1.5)	0 (1.5)
Biking (h/week)	0.5 (2.5)	1 (2.5)	0.5 (2.5)	0.8 (2.5)	1 (2.5)	0.5 (2)	0.5 (3)	0.5 (3)	0.5 (3)	1 (2.5)

Values are presented as median (interquartile range) or per cent

**Table 2:** Age- and waist circumference-adjusted correlations of height and components of height with cardiometabolic risk markers in a random subcohort (*n* = 2029) of the EPIC-Potsdam study

Variable	Men, *n* = 765				Women, *n* = 1264			
	Height	Leg length	Sitting height	LLHR	Height	Leg length	Sitting height	LLHR
FLI	−0.22	−0.25	−0.09	−0.19	−0.24	−0.24	−0.15	−0.15
Triacylglycerols	−0.11	−0.12	−0.05	−0.09	−0.12	−0.10	−0.10	−0.03
Total cholesterol	−0.12	−0.13	−0.07	−0.07	−0.10	−0.07	−0.09	−0.02
HDL-cholesterol	0.04	0.06	0.01	0.04	0.05	0.06	0.02	0.05
GGT	−0.08	−0.09	−0.04	−0.05	−0.07	−0.06	−0.06	−0.04
hsCRP	−0.02	0.01	−0.06	0.05	−0.12	−0.09	−0.12	−0.02
HbA_1c_	−0.04	−0.05	−0.05	−0.02	0	0.01	−0.01	0.01
Adiponectin	0.02	0.03	0	0.04	0.11	0.09	0.09	0.03
Systolic BP	−0.01	−0.03	0.01	−0.03	−0.11	−0.13	−0.05	−0.08
Diastolic BP	−0.03	−0.04	−0.01	−0.04	−0.07	−0.11	0	−0.10

All correlation coefficients ≥ │0.10 │ were statistically significant at *p*<0.01

hsCRP, high-sensitivity CRP; LLHR, leg length/height ratio

**Fig. 2:**
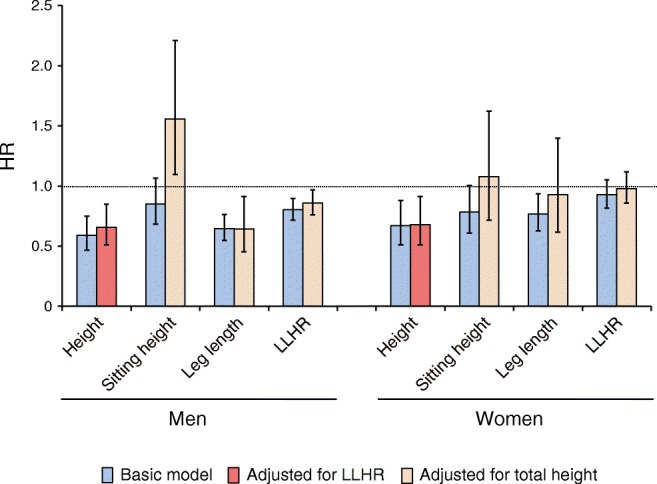
Association of height and height components with risk of type 2 diabetes in the EPIC-Potsdam study. HRs adjusted for age (stratum variable), waist circumference, education, activity, smoking and alcohol consumption. The HRs are per 10 cm greater height; per 5 cm greater sitting height and leg length; and per percentage point greater leg length/height ratio (LLHR)

Figure 2 presents associations of height and different height components with risk of type 2 diabetes, adjusting for other risk factors. Height was inversely associated with diabetes risk among men and women (HR per 10 cm, 0.59 [95% CI 0.47, 0.75] and 0.67 [0.51, 0.88], respectively). Adjustment for the leg length/height ratio had little impact on this association (HR 0.66 among men and 0.68 among women). Both larger leg length and sitting height were inversely associated with diabetes risk, although associations were non-significant for sitting height. However, if adjusted for total height, leg length remained inversely associated with risk among men while sitting height adjusted for total height was positively associated with risk. Among women, both leg length and sitting height were not meaningfully related to risk if adjusted for total height. The leg length/height ratio was inversely associated with risk among men without and with adjustment for total height, while among women this association was considerably weaker and non-significant and largely attenuated if adjusted for total height.

If we adjusted in sensitivity analyses for waist circumference accounting for the natural increase with increasing height (reflecting skeletal structure growth), the inverse association between height and diabetes risk was slightly less pronounced (HR per 10 cm, 0.70 [95% CI 0.56, 0.89] among men, 0.78 [0.60, 1.02] among women) than in our main model which included measured waist circumference (0.59 and 0.67, respectively). Furthermore, additional adjustment for family history of diabetes, smoking intensity and dietary intake of whole grains, red meat and sugar-sweetened beverages had marginal impact on the observed associations (HR per 10 cm, 0.62 among men and 0.72 among women). The association of height with diabetes risk appeared to be stronger among normal-weight individuals (HR per 10 cm, 0.14 among men, 0.33 among women) compared with overweight/obese individuals (HR, 0.64 and 0.70, respectively).

We furthermore evaluated to what extent the inverse associations of height and height components with type 2 diabetes risk are explainable by liver fat (FLI) and cardiometabolic risk factors (Table 3). Additionally, adjusting for FLI changed the HRs per 10 cm of height from 0.60 to 0.66 (−19%) among men and from 0.67 to 0.87 (−63%) among women. Among men, adjustment for HbA_1c_ and lipid variables resulted in an attenuation of the HR for height by ~10%, while GGT, CRP, adiponectin and BP had no substantial impact on the association. In contrast, among women adjustment for adiponectin (−30%) and CRP (−13%) attenuated the associations of height with diabetes, in addition to lipid markers and HbA_1c_. For relative leg length, adjusting for all biochemical markers changed the HR from 0.80 to 0.83 (−15%) and adjustment for FLI to 0.84 (−19%) among men.

**Table 3:** Mediation analysis of associations of height and height components with type 2 diabetes by cardiometabolic risk factors

Model	Women (*n*=1525, *n* cases=289)				Men (*n*=1137, *n* cases=409)							
	Height (10 cm)				Height (10 cm)				LLHR (%)			
	HR	(95% CI)	Change (%)	(95% CI)	HR	(95% CI)	Change (%)	(95% CI)	HR	(95% CI)	Change (%)	(95% CI)
Reference model^a^	0.67	(0.58, 0.77)			0.60	(0.52, 0.68)			0.80	(0.75, 0.87)		
+ Triacylglycerols	0.73	(0.63, 0.85)	−21.6	(−38.4, −12.5)	0.61	(0.53, 0.70)	−4.64	(−11.9, 0.09)	0.81	(0.76, 0.88)	−4.97	(−12.4, 0.48)
+ Total cholesterol	0.68	(0.58, 0.77)	−0.79	(−4.11, 1.00)	0.59	(0.52, 0.68)	0.62	(−2.67, 5.45)	0.80	(0.75, 0.87)	0	(−1.03, 1.50)
+ HDL-cholesterol	0.69	(0.59, 0.80)	−6.43	(−16.6, 3.66)	0.60	(0.53, 0.69)	−2.92	(−9.19, 3.61)	0.83	(0.78, 0.89)	−13.1	(−23.6, −5.08)
+ All lipid markers	0.73	(0.62, 0.84)	−20.9	(−38.1, −8.71)	0.63	(0.55, 0.72)	−9.77	(−20.7, −0.62)	0.84	(0.78, 0.91)	−19.0	(−32.7, −9.66)
+ GGT	0.69	(0.59, 0.80)	−4.82	(−18.6, 2.00)	0.60	(0.52, 0.68)	−0.99	(−2.94, 0.20)	0.80	(0.75, 0.86)	2.45	(0.42, 5.28)
+ hsCRP	0.71	(0.60, 0.82)	−12.7	(−32.2, −5.69)	0.61	(0.53, 0.69)	−2.91	(−6.56, −0.84)	0.81	(0.75, 0.87)	−1.30	(−3.60, 5.52)
+ HbA_1c_	0.70	(0.59, 0.82)	−11.6	(−33.4, 12.0)	0.63	(0.54, 0.73)	−8.93	(−26.8, 7.67)	0.84	(0.78, 0.90)	−19.1	(−42.0, −2.34)
+ Adiponectin	0.76	(0.65, 0.89)	−30.2	(−56.3, −14.9)	0.59	(0.51, 0.68)	1.60	(−6.31, 9.03)	0.79	(0.74, 0.86)	6.54	(−1.33, 17.5)
+ All biomarkers	0.81	(0.67, 0.96)	−45.6	(−84.9, −19.0)	0.63	(0.52, 0.74)	−10.7	(−30.7, 10.0)	0.83	(0.77, 0.90)	−15.0	(−40.9, 4.83)
+ BP^b^	0.68	(0.58, 0.78)	−3.26	(−14.7, 7.92)	0.59	(0.52, 0.67)	1.67	(−4.11, 8.52)	0.80	(0.74, 0.86)	3.13	(−4.05, 11.7)
+ FLI	0.87	(0.73, 1.00)	−62.9	(−105, −39.7)	0.66	(0.57, 0.75)	−19.0	(−28.5, −11.6)	0.84	(0.78, 0.90)	−18.7	(−31.1, −10.3)

The change (%) reflects the change of the estimate through additional adjustment for the indicated risk factor relative to the estimate from the reference model. Its stability as well as the corresponding HRs were estimated as median and dispersion from a bootstrapping procedure (500 bootstrap replicates)

^a^Adjusted for age (stratum variable), waist circumference, education, activity, smoking and alcohol consumption

^b^Systolic and diastolic BP and intake of antihypertensive drugs

hsCRP, high-sensitivity CRP

## Discussion

We found that in men and women the risk of future type 2 diabetes was lower by more than 30% for each 10 cm difference in height when accounting for common diabetes risk factors. For components of height, specifically leg length was inversely associated with risk in men and women. These findings are partly in line with the few previous prospective studies on height components and diabetes risk [8, 9]. In the Shanghai Women’s Health Study and the Shanghai Men’s Health Study a larger leg length was inversely related to diabetes [8]. However, this association completely diminished when adjusted for BMI. Given the negative correlation between height and body fat percentage [24, 25], adjusting for BMI may at least in part inappropriately account for beneficial effects of a higher proportion of lean body mass with larger stature. Waist circumference reflects abdominal fat accumulation and was previously found to more strongly predict diabetes risk in our cohort [1]. In our study, associations of height with diabetes risk were observed in models adjusted for waist circumference. Inverse associations were not observable for sitting height in the Shanghai Women’s Health Study [8]. In the Atherosclerosis Risk in Communities study, leg length was inversely associated with diabetes risk [9]; however, models were not adjusted for BMI or waist circumference. In this study, the inverse associations became stronger after adjustment for body weight at age 25; however, such an adjustment likely makes interpretation of height components more difficult as they may become surrogates for lean body mass. Higher leg length/height ratio was inversely associated with risk in the Atherosclerosis Risk in Communities study for both white men and women, while we observed an inverse association more clearly for men.

Our data indicate a sex difference in associations of leg length vs sitting height: a larger sitting height at the cost of leg length (sitting height adjusted for total height) was related to increased risk in men, while among women both leg length and sitting height contributed to lower diabetes risk, although the latter association was statistically non-significant. This suggests that, among boys, growth before puberty, which relates more strongly to leg length, has a more favourable impact on later diabetes risk than growth during puberty (assuming that truncal bones are last to stop growing [26]), while for girls both growth periods seem to be important. However, our observation that sitting height (not adjusting for total height) is associated with lower diabetes risk is only partly in line with other studies [8, 10, 11, 27], which makes it difficult to conclude on sex differences in relation to different growth periods from our data. While sitting height has been related to lower insulin secretory function and insulin sensitivity [10], our results indicate that a detrimental effect among men can only be expected if growth of the trunk does not result in larger total height, thus being at the cost of leg length. The positive correlation between leg length and sitting height, however, suggests that such a growth pattern might not be common in our population.

Of note, waist circumference reflects abdominal fat accumulation, but also captures general features of skeletal structure and body size and thus scales to height [28]. We addressed this point in sensitivity analyses where models were adjusted for differences in waist circumference that would not be predicted based on height alone. Height remained inversely associated with diabetes risk in these analyses, although slightly more weakly compared with models with adjustment for measured waist circumference. Also, the inverse association between height and diabetes risk was more prominent among individuals with a BMI <25 kg/m^2^. This may indicate that a higher diabetes risk with larger waist circumference counteracts beneficial effects related to height, irrespective of whether larger waist circumference is due to growth or due to an energy imbalance.

Our results furthermore indicate that a substantial proportion of the inverse association between height and diabetes risk is attributable to lower liver fat content. Specifically, in women, adjustment for the FLI substantially weakened the associations between height and diabetes risk. That taller people have lower liver fat content has been described before [4], although data on this association are rather sparse. Ectopic lipid storage strongly affects the extent of insulin sensitivity [29] and may thus be a key characteristic explaining the link between greater height and lower diabetes risk. We evaluated a variety of cardiometabolic risk factors as potential explanations of the lower diabetes risk observed with greater height. Generally, BP, triacylglycerols, CRP and adiponectin appeared to be more strongly correlated with height among women and seemed to play a stronger role in the association of height with diabetes than among men. However, the tendency towards sex differences requires confirmation. Genetic studies have not identified sexually dimorphic associations with height [30], which speaks against sex differences in potential mediators. Still, specifically blood lipids (triacylglycerol and HDL-cholesterol) consistently attenuated associations between height and diabetes risk if adjusted for. These findings are supported by studies linking genetically determined height to cardiovascular disease and several cardiometabolic risk factors, including BP, triacylglycerol and CRP levels [13, 14]. However, the role of adiponectin in this context remains unclear. Adiponectin levels rather decrease than increase during growth among children small for gestational age [31, 32]. Also, only a few genetic variants relate to both adiponectin levels [33] and height, e.g. in *LYPLAL1* and *PDE3A*. Still, it is conceivable that several overlapping and complex biologic pathways on the one hand influence height and on the other hand influence the risk of type 2 diabetes through an effect on lipid metabolism and function of adipose tissue (electronic supplementary material [ESM] Fig. 1). That these processes link height to the two key mechanisms that characterise type 2 diabetes (impaired beta cell function and insulin resistance) is supported by several studies. Tall people tend to have lower insulin resistance compared with shorter people [4, 11, 12, 24]. Growth during puberty and larger adult height have also been associated with concentrations of insulin-like growth factors [34, 35], which contribute to insulin sensitivity [36]. On the other hand, data from the Metabolic Syndrome in Men (METSIM) cohort suggest that height is also positively associated with beta cell function (disposition index) [12]. Interestingly, in this study greater height was related to improvements in both insulin sensitivity and beta cell function over time, independent of the baseline status of these two variables, age, waist circumference, physical activity and smoking. Still, whether the use of indices of insulin sensitivity and beta cell function derived from oral glucose tolerance tests in these studies is meaningful in the context of evaluation of height is questionable given that response to a fixed glucose load depends on the total amount of tissue for uptake and metabolism of glucose [37, 38]. It would be valuable to confirm such associations with gold standard methods of insulin sensitivity and secretion which account for differences in body size [39]. In our study, adjustment for HbA_1c_ attenuated the association of relative leg length with diabetes in men. However, such adjustment could be considered an overadjustment given that HbA_1c_ is used as a variable for diagnosing diabetes, although not at the time of our study. Although observational studies support an association between height and diabetes, investigation of genetically determined height has only suggested a trend for decreased risk of diabetes so far [13].

Our findings suggest that short people might present with higher cardiometabolic risk factor levels and have higher diabetes risk compared with tall people. Height can be used in diabetes risk prediction models, besides other risk factors. For example, in the German Diabetes Risk Score, points assigned per 1 cm of waist circumference or 1 year of age correspond to about 3 cm and 2 cm of height, respectively [6]. Thus, healthcare providers should be encouraged to consider height for risk assessment. On the other hand, attained height might represent an estimate of early childhood factors and their effects on later cardiometabolic risk. Thus, in terms of prevention of height-related diabetes risk, interventions likely need to focus on determinants of growth during pregnancy, early childhood, puberty and early adulthood. Although increased height was associated with reduced risk of type 2 diabetes, our data support that tallness is unlikely to modulate risk directly, but rather liver fat and other cardiometabolic risk factors are important mediators. Still, to what extent unfavourable risk factor profiles among shorter people require specific interventions remains unclear as height has not been systematically assessed as a modifying factor in this context.

The strengths of our study are that it was based on a large prospective cohort with measurements of components of stature (sitting height) and of a large variety of cardiometabolic risk factors that might explain associations of height with diabetes risk. Our study has limitations. For instance, it included only middle-aged men and women. Fat accumulation in the liver was estimated by the FLI. Although results should be replicated using more precise methods of liver fat determination, the FLI has been shown to correlate moderately with hepatocellular lipid content determined by magnetic resonance spectroscopy [40]. We have used the FLI as a proxy of liver fat content due to the absence of more precise measurements in the cohort. The FLI was strongly associated with risk of type 2 diabetes in comprehensively adjusted models (including adjustment for waist circumference) in the EPIC-Potsdam study [41]. Thus, our observations support the hypothesis that the lower risk in taller people is partially mediated by lower liver fat content. However, the adjusted model included waist circumference, an important component of the FLI, which might have led to an underestimation of the contribution of liver fat in our study. Most of the blood samples were collected non-fasting, which might have influenced specifically the interpretation of blood lipids and also the FLI in our study, which contains triacylglycerols in its calculation. However, when restricting the analysis to fasted participants, height remained inversely correlated with FLI in men (*r* = −0.22) and women (−0.15). Also, we considered only cardiometabolic markers as mediating factors being upstream from the two main pathophysiological mechanisms (insulin resistance and impaired beta cell function). Furthermore, as we do not have longitudinal data on height for our study participants, we were unable to account for height decline with ageing. Although such changes should have been small within our follow-up time [42], we cannot rule out that shorter height at study baseline has to some extent been the result of height loss prior to our study. Our analyses have been adjusted for age which should account both for age-related decline in height as well as age-cohort effects. Still, height loss has been observed to relate to higher risk of cardiovascular disease [43], but it remains unknown whether interventions to prevent age-related height decline would actually reduce diabetes risk. As for all observational studies, residual confounding might explain associations between height and diabetes risk. Specifically, parental socioeconomic status may relate to nutritional status during periods of growth and may insufficiently be reflected by participant education in our study. However, adjustment for parental socioeconomic status had little effect on associations between height and diabetes risk in the Atherosclerosis Risk in Communities study [9], and previous studies on genetic determinants of height support a biological interconnection between growth and cardiometabolic risk factors such as BP and lipid metabolism [13, 14].

In conclusion, we found an inverse association between height and risk of type 2 diabetes among men and women, which was largely related to leg length as a component of total height among men. Part of this inverse association may be driven by the associations of greater height with lower liver fat content and a more favourable profile of cardiometabolic risk factors, specifically, blood lipids, adiponectin and CRP.

## Electronic supplementary material

ESM Figure (PDF 56 kb)

## Data Availability

The datasets analysed during the current study are not publicly available due to data protection regulations. In accordance with German Federal and State data protection regulations, epidemiological data analyses of EPIC-Potsdam may be initiated upon an informal enquiry addressed to the secretariate of the Human Study Center (Office.HSZ@dife.de). Each request will then have to pass a formal process of application and review by the respective Principal Investigator and a scientific board.

## Abbreviations

CRP: C-reactive protein
EPIC: European Prospective Investigation into Cancer and Nutrition
FLI: Fatty liver index
GGT: γ-Glutamyl transferase
