# Supplementary material for: Associations of short stature and components of height with incidence of type 2 diabetes: mediating effects of cardiometabolic risk factors
Source: Diabetologia. 2019 Sep 9;62(12):2211–21. doi: 10.1007/s00125-019-04978-8 (PMC6861343; doi:10.1007/s00125-019-04978-8)
Supplement: Supplementary file 1 — (PDF 56 kb) [file 125_2019_4978_MOESM1_ESM.pdf]

ESM Fig. 1

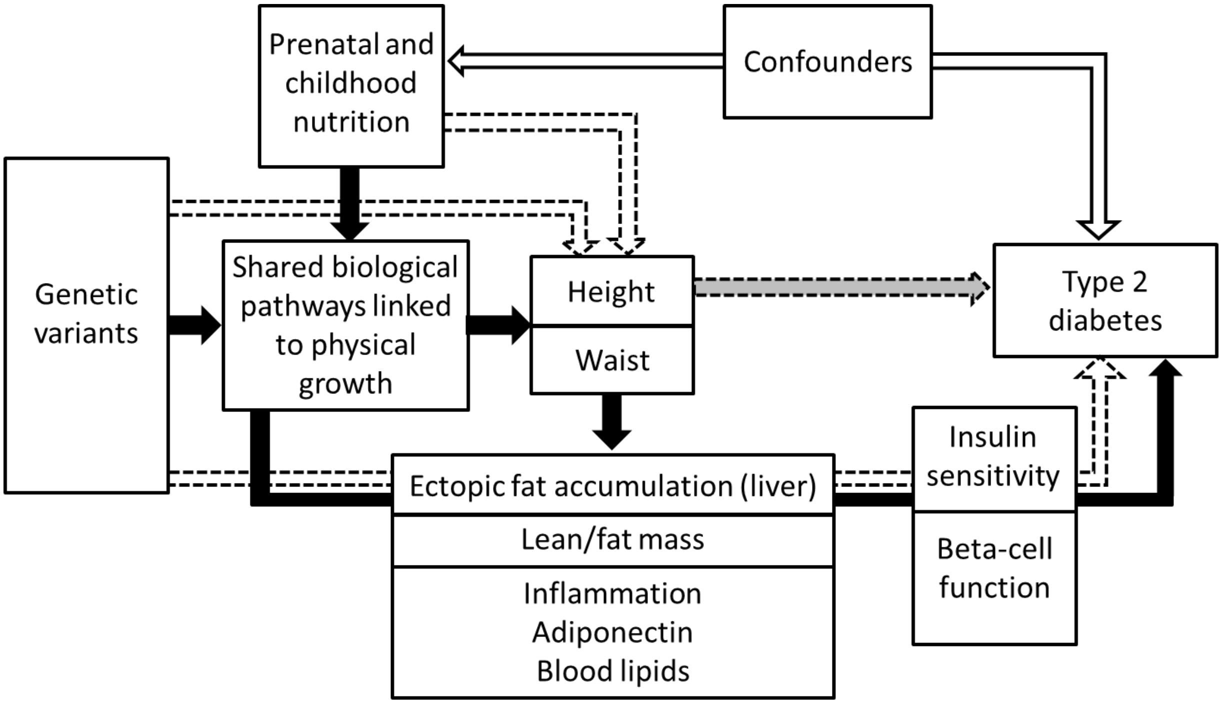

Working model for interpreting the association between shorter height and increased risk of type 2 diabetes

Genetic variants and prenatal and childhood nutrition affect biologic pathways, which on the one hand determine achieved height and on the other hand influence the risk of type 2 diabetes (solid black errors). It is also possible that height itself alters lifestyle, which then affects the risk of type 2 diabetes (dashed grey error). Physical growth will not only affect height but also waist circumference, which partly counteracts beneficial effects related to height.
